# Hair Metals and Essential Trace Element Profiles in Children With Autism Spectrum Disorder (F84.0): an Exploratory Case-Control Study With Sex Stratified Descriptive Analyses

**DOI:** 10.1007/s12011-026-05222-2

**Published:** 2026-07-09

**Authors:** Renata Rizzo, Tommaso Filippini, Chiara Copat, Alfina Grasso, Eliana Pellegrino, Valentina Crisafi, Andrea Moscato, Margherita Ferrante, Rita Barone, Maria Fiore

**Affiliations:** 1 Child and Adolescent Neurology and Psychiatry, Department of Clinical and Experimental Medicine, University of Catania, Catania, Italy; 2 Environmental, Genetics, and Nutritional Epidemiology Research Center (CREAGEN), Department of Biomedical, Metabolic and Neural Sciences, University of Modena and Reggio Emilia, Modena, Italy; 3 School of Public Health, University of California Berkeley, Berkeley, CA USA; 4 Department of Medical, Surgical Sciences and Advanced Technologies “G.F. Ingrassia”, University of Catania, Catania, 95123 Italy; 5 Department “G.F. Ingrassia” of the University of Catania, School of Medical Specialization in Hygiene and Preventive Medicine, Catania, Italy

**Keywords:** Autism spectrum disorder, Hair analysis, Trace elements, Heavy metals, Sex differences

## Abstract

This is an exploratory case–control study assessed levels of metals and essential trace elements in hair samples from children with Autism Spectrum Disorder (ASD) and neurotypical controls, exploring potential differences according to sex. A total of 154 children (61 children with ASD and 93 controls) aged 4–18 years were enrolled at the same clinical site in Eastern Sicily. Demographic, perinatal, dietary, and exposure information was collected through structured parental questionnaires. Hair samples were analyzed using inductively coupled plasma mass spectrometry. Children with ASD showed distinct elemental patterns. Females with ASD had higher median nickel (0.25 vs.0.20 µg/g), lead (1.11 vs.0.55 µg/g), chromium (0.87 vs.0.49 µg/g), and aluminum (10.27 vs. 6.97 µg/g) compared with female controls; similar but smaller differences appeared in males. Mercury levels were consistently lower in children with ASD for both sexes (females: 0.25 vs.0.60 µg/g; males: 0.30 vs.0.58 µg/g). Essential trace elements were markedly elevated in children with ASD: zinc reached 132 µg/g in ASD females vs. 83 µg/g in controls, and 129 vs. 65 µg/g in males; selenium and copper were also higher across sexes. Maternal and child characteristics differed between groups. Mothers of children with ASD reported more infections (30.9% vs. 9.6%) and therapies during pregnancy (43.9% vs.22.6%), while children with ASD showed more gastric disorders (40.4%vs.9.4%), specific diets (21.1% vs.3.5%), and vitamin use (26.3% vs.3.5%). Findings suggest sex-related differences in hair metals and trace elements profiles among children with ASD. The low mercury levels observed in children with ASD showld be interpreted with caution, given that factors such as dietary habits, supplement intake, or differences in metal metabolism were not directly assessed in this study.

## Introduction

Autism Spectrum Disorder (ASD) is a complex neurodevelopmental disorder characterized by deficits in social communication, alongside restricted and repetitive patterns of behavior and interests. The worldwide prevalence is estimated at about 1% [1]. The prevalence of ASD is significantly higher in males compared to females, with an estimated male-to-female ratio of approximately 4:1, but the underlying reasons for this disparity remain unclear [2]. The etiology of ASD is complex and multifactorial: alongside the widely recognized genetic component, environmental factors, including heavy metals and imbalance of essential trace elements, are increasingly emerging as possible contributors to ASD development and clinical variability [3–5]. Humans are continuously exposed to heavy metals through various pathways, including the ingestion of contaminated food, the inhalation of airborne particles, and, to a lesser extent, dermal absorption [6]. The diet represents one of the main sources of essential elements such as zinc (Zn) and selenium (Se) and also one of the main sources of toxic elements such as arsenic (As), lead (Pb), mercury (Hg), and cadmium (Cd) that can accumulate in fish, cereals, and vegetables grown in contaminated areas [6–9]. Metals such as lead (Pb), cadmium (Cd), mercury (Hg) are recognized for their neurotoxic potential, whereas manganese (Mn), zinc (Zn), copper (Cu) and iron (Fe) are essential for brain development and function [10]. Several biological mechanisms have been proposed to explain how metal dysregulation may contribute to ASD pathophysiology. Toxic metals such as Pb, Hg, Cd, and Cr can induce oxidative stress and mitochondrial dysfunction, processes that have been repeatedly implicated in ASD and other neurodevelopmental disorders [10, 21]. In addition, exposure to neurotoxic metals may promote neuroinflammatory responses and alter molecular pathways involved in brain development and neuronal function [3, 21]. At the same time, essential trace elements including Zn, Cu, Se, and Mn play critical roles in antioxidant defense, neurotransmission, immune regulation, and normal neurodevelopment [24, 34]. Alterations in the homeostasis of these elements during critical developmental windows may disrupt neuronal signaling and neurodevelopmental processes [24]. Increasing evidence therefore suggests that ASD may be associated not only with exposure to toxic metals but also with a broader imbalance in metal homeostasis affecting both toxic and essential trace elements [20, 24]. Moreover, Pb and Cu have attracted growing attention due to their neurotoxic properties and their ability to induce oxidative stress, immune and neuroinflammatory processes [11]. Some elements, including Pb, Hg, Fe and Mn, are capable of crossing the blood-brain barrier suggesting a possible role for metals in neurodevelopment [12]. A recent review showed that findings are not always concordant in demonstrating an association between body levels of these elements and the ASD risk [13]. Previous studies evaluating blood metal levels have frequently produced inconsistent results, especially when considering sex-related differences [14, 15]. In one study, a stratified analysis showed higher levels of lithium (Li) and Cd in children with ASD, compared to controls [15]. It is important to recognize that, under comparable exposure conditions and in absence of underlying health disorders, females tend to have essential trace elements and heavy metal levels that differ from those of males [16]. Based on the above considerations, the aim of our study was to describee and compare hair metal and essential trace element levels in children with ASD and neurotypical controls, with particular attention to potential sex-related differences.

## Materials and Methods

### Study Design, Setting and Participants

We conducted an exploratory a case–control study at the Child Neuropsychiatry Unit, University Hospital “Policlinico G. Rodolico-San Marco” in Catania (Sicily, Italy). Given the exploratory nature of the study, no formal sample size calculation was undertaken. Instead, all eligible children fulfilling the predefined inclusion and exclusion criteria and recruited during the study period were included in the analysis. Accordingly, the study was not designed to achieve a predefined statistical power, and the findings should be interpreted as descriptive and hypothesis-generating Patients included children diagnosed with ASD (F84.0) between 2010 and 2020, all born and residing in Eastern Sicily. Controls were recruited at the same geographical area and enrollment site as the patients and included individuals aged 4 to 18 years, of both sexes, with no diagnosed medical conditions.

To limit the influence of potential confounding factors on trace element levels, participants with additional neuropsychiatric conditions (such as cerebral palsy, ICD-10: G80–G83), eating disorders (ICD-10: F50), a vegetarian diet, endocrine-related illnesses (including obesity and diabetes), metal implants (such as dental amalgams), recent physical trauma, inflammatory diseases, or those using shampoos with mineral additives or hair care products were excluded from the study. Additionally, all selected children lived in areas without proximity to industrial pollution sources. The study protocol received approval from the Ethics Committee (Approval Number 118/CEL 21 May 2024) of the University Hospital Policlinico “G. Rodolico – San Marco” in Catania. All procedures complied with the ethical guidelines outlined in the Declaration of Helsinki (1964) and its later revisions (2013). Before enrollment, a written informed consent was obtained from the parents of all participating children.

### Diagnostic Criteria

ASD diagnosis was based on the Diagnostic and Statistical Manual of Mental Disorders, Fifth Edition (DSM-5) criteria [17], supported by Autism Diagnostic Interview–Revised (ADI-R) and Autism Diagnostic Observation Schedule (ADOS).

### Data Collection

Information on demographic, perinatal, clinical, lifestyle, and dietary factors was collected using a structured questionnaire administered to parents by trained personnel. The questionnaire was specifically developed for the study and structured into thematic sections aimed at capturing variables potentially associated with metal and essential trace element levels in hair, as well as relevant prenatal and early-life exposures. The first section collected parental socio-demographic characteristics (age, education level, occupation, marital status) and information on possible occupational or domestic exposures. A dedicated section investigated maternal conditions during pregnancy, including infections, therapies, smoking and alcohol habits, use of supplements or herbal products, and dietary patterns (e.g., daily intake of fruits and vegetables, weekly fish consumption, type of water consumed). A second section focused on the child’s characteristics, including sex, age, type of delivery, feeding practices during infancy (breastfeeding, formula, mixed feeding), previous diseases, therapies, and gastrointestinal or infectious conditions. Particular attention was given to factors potentially influencing hair metal levels, such as the use of cosmetic hair products containing minerals (dyes, gels, oils), presence of dental fillings, and piercing use. Finally, a dietary section focused on current child nutrition and water consumption, including the use of filtered, tap, or mineral water, daily intake of fruits and vegetables, weekly fish consumption, adherence to specific diets, and the use of vitamins or dietary supplements.

### Collection, Preparation, and Chemical Determination of Metals and Essential Trace Elements in Hair Samples

Hair samples were selected as the biological matrix because they provide a non-invasive indicator of medium- to long-term exposure to metals and trace elements, reflecting accumulation over several weeks to months. Compared with blood or urine, hair collection is simple, well tolerated in pediatric populations, and allows the simultaneous assessment of multiple elements. Furthermore, hair biomonitoring has been widely used in environmental epidemiology and neurodevelopmental research to investigate exposure patterns and alterations in metal homeostasis. To minimize the influence of external contamination, standardized collection procedures and a washing protocol were applied prior to analysis. Hair samples were obtained by cutting the proximal portion of occipital hair strands using stainless steel scissors previously cleaned with ethanol, with a target amount of approximately 1–2 g per subject. Samples were consistently collected from the proximal occipital region; however, the exact length of the analyzed hair segment was not systematically recorded. Consequently, although hair was used as a biomarker of medium- to long-term exposure, the specific exposure window represented by each sample cannot be more precisely defined than several weeks to months. Each specimen was immediately placed in labeled polypropylene tubes indicating participant code, collection date, and time. Prior to analysis, hair samples underwent a decontamination process consisting of three washes with double-distilled water, followed by drying in an oven at 60 °C until constant weight. Currently, no internationally accepted standard exists for hair washing prior to elemental analysis. Different protocols, including acetone-, detergent-, water-, or mixed-based procedures, have been adopted in the literature, each with specific advantages and limitations regarding the removal of exogenous contamination and the preservation of endogenous elemental content [18, 19]. Therefore, the washing procedure used in this study followed the validated analytical protocol routinely implemented in our laboratory for ICP-MS analysis and was applied consistently to all samples. For mineralization, an aliquot of 0.5 g of dried hair was digested in a closed-vessel microwave system (Ethos TC, Milestone; Sorisole, Italy) using acid-cleaned TFM containers. The digestion mixture consisted of 6 mL of ultrapure nitric acid (65% w/v; Merck, Darmstadt, Germany) and 2 mL of hydrogen peroxide (30% w/v; Sigma-Aldrich, St. Louis, USA). The microwave program included three sequential steps: 15 min ramp to 180 °C (1000 W), 15 min hold at 180 °C (1000 W), and 15 min cooling. Alongside the samples, reagent blanks prepared with the same acid mixture were processed identically. Following digestion and cooling, solutions were transferred into 50 mL Falcon^®^ tubes and diluted with double-distilled water to the final volume. The quantification of metals and essential elements was performed using inductively coupled plasma mass spectrometry (ICP-MS; Elan DRC-e, Perkin Elmer, USA). Calibration curves were obtained from multi-element standard solutions (0.5–100 µg/L; CPAchem, ISO17034 and ISO/IEC 17025 accredited). Yttrium (Y) and Rhenium (Re), prepared at 50 µg/L from single-element standards (CPAchem, ISO17034 and ISO/IEC 17025 accredited), were used as internal standards for online correction of instrumental drift. Analytical quality assurance included the use of laboratory-fortified matrices for each digestion batch. Mean recovery rates for all analyzed elements remained between 90% and 110% throughout the study period. Elements of interest included Li, Be, Al, Cr, Co, Mn, Ni, Zn, Cu, As, Se, Mo, Cd, Hg, U, Pb.

### Statistical Analysis

Statistical analyses were intentionally limited to descriptive estimation, consistent to the principles advocated by the American statistical Association (ASA), which emphasize transparent reporting of the observed data and avoidance of dichotomous statistical significance testing. Continuous variables were summarized within each study group using medians and corresponding 95% confidence intervals to describe the central tendency and precision of each distribution. No formal between-group effect estimators (e.g., median differences or ratios of medians) were calculated because the objective of this exploratory study was to characterize distributional patterns of metal and trace element concentrations rather than to estimate comparative effects. Accordingly, confidence intervals are presented solely to measures of precision for the within-group estimates and are not interpreted as evidence for between-group differences. Confidence intervals for the median were estimated using the exact distribution-free approach based on binomial order statistics. For measurements below the limit of quantification (LOQ), values were imputed as LOQ/2 to allow their inclusion in the statistical analyses. Stratified analyses were conducted separately for patients and controls by sex. All statistical analysis were performed by Statistical Package for Social Sciences, version 30, software (SPSS Inc., Chicago, IL, USA)..

 [20, 21].

## Results

A total of *154* children were included in the study (*61* ASD patients and *93* controls).

### Demographic Characteristics and Dietary Habits of Mothers

Maternal socio-demographic and exposure-related characteristics are summarized in Table 1. Differences in maternal characteristics were observed between groups. Mothers of children with ASD reported a larger observed frequency of infections during pregnancy (30.9% vs. 9.6%) and more frequent use of therapies during gestation (43.9% vs. 22.6%). Educational level also differed, with a lower proportion of university graduates (17.5% vs. 37.3%) and a higher proportion of housewives (62.3% vs. 39.8%) in the ASD mother group. Other variables, including smoking habits, marital status, blood type distribution, and most lifestyle factors, were largely comparable between groups.

**Table 1:** Demographic characteristics of mothers

Variables	Children with ASD *N* = 61 *n* (%)	Controls *N* = 93 *n* (%)
Smoking mother*	12 (21)	17 (20)
Number cigarettes	9 (5–15)	10 (8–15)
Smoking during pregnancy	3 (5)	2 (2)
Marital status		
*Married*	51 (89.5)	72 (86.7)
*Cohabitant*	2 (3.5)	8 (9.6)
*Divorced*	4 (7)	3 (3.6)
Education level		
*High School Diploma*	33 (57.9)	35 (42.2)
*Degree*	10 (17.5)	31 (37.3)
*Elementary School Diploma*	1 (1.8)	1 (1.2)
*Lower Middle School Diploma*	13 (22.8)	16 (19.3)
Profession		
*Housewife*	38 (62.3)	37 (39.8)
Other**	23 (37.7)	56 (60.2)
Blood type		
*0 Rh-*	3 (5.4)	3 (3.8)
*0 Rh+*	24 (42.9)	35 (43.8)
*A Rh-*	1 (1.8)	4 (5)
*A Rh+*	21 (37.5)	26 (32.5)
*AB Rh+*	4 (7.1)	4 (5)
*B Rh+*	3 (5.4)	7 (8.8)
*B Rh-*	0 (0)	1 (1.3)
Anti-D Ig (yes)	0 (0)	3 (3.8)
Flu vaccine during pregnancy (yes)	0 (0)	2 (2.4)
Infections during pregnancy (yes)	17 (30.9)	8 (9.6)
Diseases**(yes)	22 (40)	24 (29.3)
Therapies during pregnancy ***(yes)	25 (43.9)	19 (22.6)
Hobbies (yes)	4 (7)	14 (16.7)
Hair products (yes)		
*Hair Dye*	18 (31.6)	21 (25)
*Hair Gel*	1 (1.8)	1 (4.8)
*Oils*	0 (0)	8 (9.5)
Piercing (yes)	5 (8.8)	3 (3.7)
Dental filling (yes)	24 (42.1)	34 (40.5)

Values are reported as number (percentage) unless otherwise specified

* Due to missing data, percentages for smoking-related variables were calculated using participants with available information as the denominator. Smoking habits were not reported by 13 participants (cases and controls combined)

**Other: Architect, Lawyer, Shop Clerk, Accountant, Insurance Consultant, Unemployed, Journalist, Employee, Nurse, Engineer, Teacher, Freelancer, Cleaning Operator, Social Health-Care Assistant, Hairdresser, Pedagogist, Bookkeeper, Secretary, Veterinarian. ** Allergy (13), Chronic migraine (1), Iron-deficiency anemia and polycystic ovary (1), Rheumatoid arthritis (2), Asthma (2), Gallbladder stones (2), Celiac disease (1), Gestational diabetes (3), Anxiety disorder (1), Migraine and psoriasis (1), Hypertension (3), Hyperthyroidism (2), Hyperthyroidism following second pregnancy (1), Hypothyroidism (4), Hodgkin’s lymphoma and recurrent iron-deficiency anemia (2), Thyroid nodules (1), Thyroid disease with thyroidectomy (2), Previous thyroidectomy (1), Early-stage psoriasis in thyroidectomized patient (1), Thrombophilia (4). *** Antihypertensive (3), Antacids (1), Antidepressant/anxiolytic (1), Antihistamines (4), Insulin (2), Biochetasi (1), Cardioaspirin, heparin, vasosuprina (1), Cardiospirin (1), Heparin (5), Heparin, Progynova, Prontogest, Progeffik, Systen (1), Euthyrox (6), Tocolytic (11), Progesterone (2)

Table 2 shows mothers’ water consumption and dietary habits. Maternal dietary habits showed several noteworthy differences. Mothers of ASD patients reported a less frequent daily consumption of fruits (42.1% vs. 59.5%) and vegetables (52.6% vs. 67.9%) and a lower weekly intake of fish (28.1% vs. 44%) compared with control mothers. Mineral water consumption was also slightly less common in the ASD group (86% vs. 96.3%). Conversely, the use of dietary supplements during pregnancy was more frequently reported among mothers of ASD patient group (63.2% vs. 46.4%). Other variables, including alcohol intake and the use of herbal products, were similar between groups.

**Table 2:** Mothers’ water consumption and dietary habits during pregnancy

Variables*	Children with ASD *N* = 61 *n* (%)	Controls *N* = 93 *n* (%)
Water		
*Tap Water*	1 (1.8)	1 (1.2)
*Mineral Water*	49 (86)	78 (96.3)
*Tap and Mineral Water*	5 (8.8)	2 (2.5)
*Filtered Water*	2 (3.5)	0 (0)
Fruits (daily consumption)	24 (42.1)	50 (59.5)
Vegetables (daily consumption)	30 (52.6)	57 (67.9)
Fish (weekly consumption)	16 (28.1)	37 (44)
Alcohol (daily consumption)	1 (1.8)	1 (1.2)
Dietary supplements	36 (63.2)	39 (46.4)
Herbal products	2 (3.5)	3 (3.6)

Percentages were calculated using the number of participants with available data for each variable. Therefore, denominators may vary because of missing data

*Only the “yes” responses were reported

### Demographic and Clinical Characteristics of Patients and Controls

The sociodemographic and clinical characteristics of the children included in the study are reported in Table 3. The ASD group shows a clear predominance of males (70.2% vs. 30.6% in controls), while females were markedly more represented in the control group. Median age was comparable between groups, with only minimal differences in both males and females. Cesarean delivery was more common in the ASD group (59.7% vs. 50%), although the difference was moderate. Different distributional patterns were observed in feeding practices: breastfeeding was less frequent in ASD (36.8% vs. 52.9%), whereas mixed feeding was more common (28.1% vs. 14.1%).

**Table 3:** Sociodemographic and clinical characteristics of patients and controls

Variables*	Children with ASD *N* = 61 *n* (%)	Controls *N* = 93 *n* (%)
Sex		
*Male*	40 (70.2)	26 (30.6)
*Female*	17 (29.8)	59 (69.4)
Median age years (IQR)		
*Male*	6.0 (3.3–9.5)	6.5 (3.8–10.3)
*Female*	5.0 (3.0–8.5)	7.0 (4–10)
Type of birth		
*Cesarean Section*	34 (59.7)	42 (50)
*Natural Birth*	23 (40.4)	42 (50)
Breastfeeding		
*Breastfeeding*	21 (36.8)	45 (52.9)
*Bottle Feeding*	20 (35.1)	28 (32.9)
*Mixed Feeding*	16 (28.1)	12 (14.1)
Hypoxia	6 (10.5)	6 (7.1)
Chronic Bacterial Infections	2 (3.5)	3 (3.5)
Dental Filling	1 (1.8)	0 (0)
Diseases (celiac disease, epilepsy, thyroid diseases)	14 (24.6)	16 (18.8)
Gastric Disorders	23 (40.4)	8 (9.4)
Intestinal Permeability Disorders	1 (1.8)	0 (0)
Therapies**	12 (21.8)	11 (12.9)

Values are reported as number (percentage) unless otherwise specified

Percentages were calculated using the number of participants with available data for each variable. Therefore, denominators may vary because of missing data

* Only the “yes” responses were reported

**Antihistamine (8), Immunostimulant (4), Bronchodilator (1), Antacid (3), Laxative (2), Antidepressant (1), Melatonin (1), Contraceptive pill for acne (1)

Regarding clinical conditions, distinct distributional patterns were observed for gastric disorders, which were reported in 40.4% of children with ASD and 9.4% of controls. Other variables, including hypoxia, chronic bacterial infections, previous diseases, and therapies, show smaller differences between the two groups.

### Dietary Habits, Water Consumption, and Supplement Use in Patients and Controls

Dietary habits, water consumption, and supplement use in ASD patients and controls are summarized in Table 4. Mineral water consumption was slightly lower in patients compared to controls (87.7% vs. 96.5%), with a modestly higher use of filtered water among patients with ASD. Dietary habits showed more marked differences: specific diets were notably more common in the ASD group (21.1% vs. 3.5%), as was the use of dietary supplements (14% vs. 4.7%). A substantial difference also emerged in vitamin intake, reported by 26.3% of patients compared with only 3.5% of controls. Other dietary variables, including fruit, vegetable, and fish consumption, showed smaller variations between the two groups.

**Table 4:** Dietary habits, water consumption, and supplement use in patients and controls

Variables*	Children with ASD *N* = 61 *n* (%)	Controls *N* = 93 *n* (%)
Water		
*Tap Water*	2 (3.6)	1 (1.2)
*Mineral Water*	50 (87.7)	82 (96.5)
*Tap and Mineral Water*	2 (3.5)	1 (1.2)
*Filtered Water*	3 (5.3)	1 (1.2)
Fruits (daily consumption)	26 (45.6)	44 (51.8)
Vegetables (daily consumption)	18 (31.6)	35 (41.2)
Fish (weekly consumption)	16 (28.1)	35 (41.2)
Specific diet**	12 (21.1)	3 (3.5)
Dietary supplements	8 (14)	4 (4.7)
Vitamins	15 (26.3)	3 (3.5)

Values are expressed as number (percentage)

Percentages were calculated using the number of participants with available data for each variable. Therefore, denominators may vary because of missing data

*Only the “yes” responses were reported. **Diet free from: gluten (8), lactose (9), casein (2), and fish (1)

### Distribution of Metal and Essential Trace Element Levels (μg/g) in Hair Samples From Asd Patients and Controls, Stratified by Sex

Hair metal and essential trace element levels showed heterogeneous patterns when comparing children with ASD (F84.0) and neurotypical controls, with some indications of sex-related differences (Table 5).

**Table 5:** Metal and essential trace element levels (µg/g)

Variables	Children with ASD median (IQR*) 95% confidence interval		Controls median (IQR*) 95% confidence interval	
	Female	Male	Female	Male
	*n* = 17	*n* = 40	*n* = 59	*n* = 26
Metals				
Nickel	0.25 (0.18–0.56)	0.09 (0.05–0.31)	0.20 (0.12–0.33)	0.10 (0.03–0.20)
	0.17–0.51	0.08–0.17	0.14–0.25	0.05–0.15
Lead	1.11 (0.44–2.26)	0.58 (0.30–1.67)	0.55 (0.28–1.20)	0.84 (0.67–1.65)
	0.44–1.86	0.46–0.97	0.36–0.87	0.69–1.46
Cadmium	0.02 (0.01–0.04)	0.01 (0.01–0.03)	0.02 (0.01–0.03)	0.02 (0.01–0.03)
	0.01–0.04	0.01–0.02	0.01–0.02	0.01–0.02
Mercury	0.25 (0.13–0.81)	0.30 (0.07–0.86)	0.60 (0.26–1.56)	0.58 (0.16–1.13)
	0.14–0.62	0.15–0.53	0.53–0.78	0.21–1.07
Chromium	0.87 (0.53–1.77)	1.02 (0.53–1.42)	0.49 (0.38–0.71)	0.51 (0.40–0.72)
	0.53–1.74	0.69–1.24	0.43–0.54	0.42–0.56
Lithium	0.03 (0.03–0.03)	0.03 (0.03–0.03)	0.03 (0.03–0.03)	0.03 (0.03–0.03)
	0.03.0.03	0.03–0.03	0.03–0.03	0.03–0.03
Beryllium	0.03 (0.00–0.03)	0.03 (0.00–0.03)	0.03 (0.00–0.03)	0.03 (0.00–0.03)
	0.03–0.03	0.03–0.03	0.03–0.03	0.03–0.03
Aluminum	10.27 (6.14–15.05)	6.95 (4.97–9.50)	6.97 (4.69–10.80)	6.98 (3.87–11.20)
	7.73–14.17	5.89–8.03	5.82–9.39	5.78–9.78
Arsenic	0.06 (0.03–0.15)	0.08 (0.05–0.15)	0.04 (0.04–0.07)	0.07 (0.05–0.09)
	0.03–0.11	0.05–0.14	0.04–0.05	0.05–0.09
Uranium	0.06 (0.05–0.10)	0.04 (0.03–0.05)	0.03 (0.03–0.05)	0.04 (0.03–0.08)
	0.06–0.10	0.03–0.05	0.03–0.03	0.03–0.06
Essential trace elements				
Manganese	0.19 (0.06–0.43)	0.12 (0.05–0.25)	0.13 (0.05–0.18)	0.12 (0.04–0.19)
	0.07–0.36	0.07–0.18	0.09–0.16	0.07–0.17
Zinc	132 (76–218)	129 (80–156)	83 (56–128)	65 (48–111)
	94–212	110–146	65–96	55–96
Copper	11.42 (8.09–16.60)	8.23 (6.79–10.83)	10.12 (6.90–14)	7.19 (5.39–9.31)
	8.27–15.50	7.70–9.24	8.40–12.10	6.30–8.90
Selenium	0.49 (0.42–0.57)	0.51 (0.44–0.60)	0.40 (0.36–0.47)	0.44 (0.36–0.50)
	0.43–0.56	0.46–0.56	0.38–0.44	0.38–0.48
Cobalt	0.03 (0.03–0.11)	0.03 (0.03–0.03)	0.03 (0.03–0.03)	0.03 (0.03–0.03)
	0.03–0.10	0.03–0.03	0.03–0.03	0.03–0.03
Molybdenum	0.09 (0.03–0.16)	0.05 (0.03–0.09)	0.05 (0.03–0.07)	0.05 (0.03–0.09)
	0.03–0.14	0.03–0.07	0.04–0.06	0.04–0.07

*IQR: interquartile range (25th-75th)

#### Metals

Among the analyzed metals, Cr, Pb, Hg, and Ni exhibited the most distinctive distributional patterns across the study groups.

For Cr, both female and males with ASD showed higher median values compared with controls. Females with ASD exhibited a median of 0.87 (0.53–1.74) versus 0.49 (0.43–0.54) in controls, while males with ASD had a median of 1.02 (0.69–1.24) compared with 0.51 (0.40–0.72) male controls. Higher median Cr levels were observed in children with ASD than in controls in both sexes.

Pb levels tended to be higher in children with ASD, particularly among females, whose median (1.11; 0.44–1.86) exceeded that of female controls (0.55; 0.36–0.87). In males, Pb levels were more similar between groups, and the estimates showed substantial variability.

Hg levels showed lower or comparable medians in children with ASD compared to controls: females with ASD had 0.25 (0.14–0.62) versus 0.60 (0.53–0.78) in controls, and males with ASD 0.30 (0.15–0.53) versus 0.58 (0.21–1.07) in controls. Median Hg levels were lower in children with ASD than in controls in both sexes, although substantial variability was observed.

Higher median Ni levelss were observed among females with ASD (0.25 µg/g; 95% CI: 0.17–0.51) compared with female controls (0.20; 0.14–0.25), whereas males with ASD showeded levelslevels similar to those of male controls (0.09; 0.08–0.17 vs. 0.10; 0.05–0.15).

Al median levels were higher in females with ASD (10.27; 7.73–14.17) than in female controls (6.97; 5.82–9.39). In contrast, males showed very similar values across groups.

For As, male and females with ASD showed slightly higher medians than controls, but with considerable variability in the estimates.Uranium (U) appeared marginally higher in females with ASD (0.06; 0.06–0.10) compared with female controls (0.03; 0.03–0.03), whereas male levels were similar between males with ASDA and male controls.

#### Essential Trace Elements

Among essential trace elements Zn, Cu and Se displayed distinct distribution patterns across the study groups.For Zn, the observed median concentration was 132 µg/g(95% CI: 94–212) in female with ASD and 83 µg/g (95% CI: 65–96) in female controls. In males, the corresponding medians were 129 µg/g (95% CI: 110–146) and 65 µg/g (95% CI: 55–96), respectively.For Cu, the observed median concentration was 11.42 µg/g (95% CI: 8.27–15.50) in female with ASD and 10.12 µg/g (95% CI: 8.40–12.10) in female controls. The corresponding median concentrations in males were 8.23 µg/g (95% CI: 7.70–9.24) and 7.19 µg/g (95% CI: 6.30–8.90), respectively. The observed differences appeared modest but directionally consistent.

For Se, the observed median concentration was 0.49 µg/g (95% CI: 0.42–0.57) in females with ASD and 0.40 µg/g (95% CI: 0.36–0.47) in female controls. In males, the corresponding median concentrations were 0.51 µg/g (95% CI: 0.44–0.60) in children with AD and 0.44 µg/g (95% CI: 0.36–0.50) in controls. The observed distributions were broadly comparable between females and males. For Mn, the observed media concentration was 0.19 µg/g (95% CI 0.06–0.43) in females with ASD and 0.13 µg/g (95% CI 0.05–0.18) in female controls. In males, the corresponding median concentrations were 0.12 µg/g (95% CI 0.05–0.25) in children with ASD and 0.12 µg/g (95% CI 0.04–0.19) in controls. Confidence intervals indicated substantial variability in the within-group estimates. For Mo, the observed median concentration was 0.0 µg/g (95% CI 0.03–0.16) in females with ASD and 0.05 µg/g (95% CI 0.03–0.07) in female controls. In males, the corresponding median concentrations were 0.05 µg/g (95% 0.03–0.09) in children with ASD and 0.05 µg/g (95% 0.03–0.09) in controls. modest.

## Discussion

In this exploratory case–control study evaluating a broad panel of metals and essential trace elements in hair, we observed heterogeneous patterns between children with ASD and neurotypical controls, with notable sex-specific differences. Such variability aligns with the broad heterogeneity reported in previous environmental studies on ASD, where results often differed according to biological matrix, analytical methods, and population characteristics [3, 22, 23].

The observed distributions of Ni, Pb, Cr, Al, Zn, Cu and Se differed across the study groups, particularly among females. This observation is coherent with several studies reporting altered metal homeostasis in ASD. For example, Skalny et al. found increased hair levels of multiple metals, including Zn, Cu and Pb, among ASD children, with significant modulation by sex and age [24]. Skogheim et al. analyzed the levels of numerous metals and essential elements in maternal blood samples collected during pregnancy and observed that specific patterns of prenatal exposure were associated with an increased risk of ASD or ADHD in the offspring. In particular, the study showed that alterations in the levels of essential elements (such as Mn, Zn, and Cu) and potentially toxic metals (such as Pb and Cd) were related to differences in neuropsychiatric outcomes in children. The authors suggest that these associations reflect an early dysregulation of metal homeostasis, already occurring during fetal development, and that this dysregulation may influence neurobiological processes critical for brain development [25]. The observed Cr concentrations, as detected in our male and female ASD groups, are consistent with evidence linking chromium exposure to oxidative stress, impaired neurotransmission, and neurobehavioral effects relevant to ASD pathophysiology [10, 26, 27]. Likewise, the observed Pb concentrations, especially in females, reflect well-known neurodevelopmental risks associated with lead exposure and its ability to cross the blood–brain barrier and induce neuroinflammation [11, 12].

Conversely, the observed median Hg concentrations were numerically lower in children with ASD across both sexes. Similar findings have been reported in previous studies, although the underlying mechanisms remain uncertain. Several hypotheses have been proposed, including differences in metal metabolism, detoxification pathways, and metal excretion patterns in children with ASD [26, 28, 29]. In our study the reported frequency of supplement use was greater among children with ASD compared with controls; however, the specific composition of supplements and biomarkers of metal-handling pathways were not available. Therefore, any potential role of zinc supplementation, metallothionein induction, or altered mercury metabolism should be considered speculative and warrants further investigation in studies specifically designed to address these mechanisms. One potential explanation proposed in the literature involves zinc-dependent modulation of metallothionein activity. Zinc is a strong inducer of metallothioneins (MTs), cysteine-rich intracellular proteins involved in the detoxification and sequestration of metals such as Hg²⁺, Cd²⁺, Cu²⁺ and Zn²⁺ [30]. Increased zinc intake may upregulate MTs expression, enhancing intracellular retention of Hg and reducing its incorporation into hair keratin. This mechanism is supported by experimental studies showing that prior zinc exposure or MTs induction protects against subsequent toxicity of heavy metals and modifies their tissue distribution and excretion patterns [31, 32]. Thus, the lower hair Hg in ASD patients does not necessarily indicate lower exposure, but rather a shift in mercury handling, as also suggested by literature on glutathione-dependent detoxification and redox imbalance in ASD [10, 26, 29, 33].

Regarding essential trace elements, the observed distributions of Zn, Cu and Se differed across the study groups. While some earlier hypotheses focused on deficiencies of essential elements in ASD, more recent hair and blood analyses report elevated or dysregulated levels, interpreted as markers of altered metabolism or homeostatic compensation [24–26, 29]. The observed Zn and Cu distributions are consistent with evidence suggesting dysregulation of the Zn/Cu ratio in ASD, which has been linked to impaired metallothionein function, oxidative stress and immune activation [26, 34]. The observed Se concentrationsare also consistent with studies highlighting altered selenoprotein activity and antioxidant responses, given selenium’s role in mitochondrial protection and redox regulation [35, 36].

The sex-specific patterns observed, particularly for Ni, Pb, Cr, Al and U, mirror the literature emphasizing sexually dimorphic responses to metal exposure. Vahter et al. demonstrated that sex modulates the absorption, distribution and toxicity of metals, partly due to differences in iron stores, hormonal influences and expression of metal transporters [16]. More recently, both mechanistic and epidemiological studies have reinforced the idea that sex differences are crucial in metal-induced diseases and neurodevelopmental outcomes, including ASD and ADHD [14, 25, 37, 38]. Our findings support further exploration of sex-stratified analyses, as pooling data across sexes can obscure meaningful associations, especially in ASD where male predominance and biological dimorphism coexist.

Taken together, these descriptive observations are consistent with the hypothesis that ASD may involve a broader dysregulation of metal homeostasis, affecting both essential and toxic elements. Metals such as Pb, Cr and Ni can induce oxidative stress, mitochondrial dysfunction, neuroinflammation and epigenetic alterations, mechanisms widely implicated in ASD neurobiology [3, 10, 11, 23, 26, 27, 36]. At the same time, perturbations in essential elements such as Zn, Cu and Se may reflect compensatory metabolic responses or downstream effects of altered metal-handling proteins, including metallothioneins and selenoproteins [26, 30, 34–36]. Our findings are in line with reviews pointing to trace element imbalances and difficulties in eliminating toxic metals as potential contributors to ASD symptom severity [22, 26, 39].

While these observations are broadly consistent with previous reviews, the available evidence on metal and trace element levels in ASD remains heterogeneous and, in some cases, contradictory. De Palma et al. reported no clear correlation between hair metal levels and autism, suggesting that hair elemental profiles alone may not reliably distinguish children with ASD from neurotypical controls [15]. Similarly, a meta-analysis by Guo et al. did not identify significant differences in hair lead levels between ASD cases and controls, highlighting the variability of findings across studies [40]. In addition, findings from Jordanian pediatric populations support the relevance of hair-based biomonitoring in neurodevelopmental research. Rashaid et al. reported altered heavy metal and trace element levels in scalp hair samples of children with severe ASD compared with controls [41]. Similarly, Bashtawi et al. observed differences in essential minerals and heavy elements in scalp hair samples of children with specific language impairment compared with fluent controls, suggesting that trace element dysregulation may also be relevant across broader neurodevelopmental conditions [42]. Furthermore, Majewska et al. observed age-dependent differences in hair mercury levels, with both lower and higher levels reported among children with ASD depending on the age group considered [28]. Consistent with these observations, a recent systematic review and meta-analysis by Ding et al. highlighted substantial heterogeneity across populations, geographical regions, biological matrices, and exposure assessment methods [43]. Taken together, these findings suggest that the relationship between metal homeostasis and ASD is complex and likely influenced by multiple biological, environmental, and methodological factors. Therefore, our results should be interpreted cautiously and viewed as contributing to a growing but still inconsistent body of evidence regarding trace element dysregulation in ASD.

Our study has several strengths, including strict criteria for sample collection to minimize external contamination, detailed assessment of dietary and perinatal factors, ICP-MS measurement of a wide metal panel, and sex-stratified analyses. However, limitations must be acknowledged: the case–control design restricts causal inference; hair levels reflect exposure and metal handling rather than internal target-organ levels and may be influenced by residual external contamination despite standardized washing procedures. Therefore, the observed distributions should be interpreted as indicators of altered exposure patterns and metal homeostasis rather than direct evidence of metal levels within the central nervous system. Sample size, particularly in female subgroups, limits precision of estimates. Additionally, although hair samples were consistently collected from the proximal occipital region, the exact length of the analyzed hair segment was not systematically recorded. Because scalp hair grows at an average rate of approximately 1 cm per month, the absence of precise segment-length information prevents a more accurate definition of the exposure window represented by each sample. Accordingly, hair measurements should be interpreted as indicators of cumulative medium-term exposure rather than exposure during a precisely defined time interval. Another limitation is the absence of multivariable adjustment for potential confounding factors. Although information on several maternal, dietary, lifestyle, and clinical characteristics was collected, the primary objective of this study was exploratory and descriptive, focusing on the characterization of metal and trace element profiles in children with ASD and controls by sex. Moreover, the relatively limited sample size, particularly after sex stratification, constrained the feasibility of building stable multivariable models including multiple covariates. Consequently, the findings should be interpreted as descriptive observations and hypothesis-generating rather than evidence of independent effects or causal relationships. In addition, children with ASD reported a higher frequency of vitamin and dietary supplement use than controls. Because detailed information regarding supplement composition, dosage, and duration was not available, the potential influence of supplementation on trace element levels, particularly Zn, Cu, and Se, could not be adequately assessed. Furthermore, detailed information on specific sources of aluminum exposure, including the use of aluminum cookware, proximity to industrial emission sources, vaccine history, and other environmental exposure pathways, was not systematically collected. Therefore, the contribution of these factors to the observed aluminum levels could not be evaluated. Moreover, biomarkers of metal-handling pathways (e.g., metallothioneins, glutathione, selenoproteins) were not available, preventing direct testing of some mechanistic hypotheses raised here.

### Potential Clinical and Research Implications

The present findings do not support direct clinical applications, they contribute to the growing body of evidence suggesting that alterations in metal and trace element homeostasis may be associated with ASD. The observed distributional patterns in hair elemental profiles warrant further investigation in larger longitudinal studies to clarify their temporal relationship with ASD and their potential biological relevance. Future research integrating environmental exposure assessment, nutritional factors, biomarkers of metal metabolism, and clinical outcomes may help determine whether specific trace element patterns could eventually contribute to a more comprehensive understanding of ASD pathophysiology.

## Conclusion

Overall, our findings describe distribution of metal and trace element concentrations in hair samples obtained fromchildren with ASD and neurotypical controls. These observations should be interpreted as descriptive and hypothesis-generating rather than as evidence of comparative effects or associations. Future research should integrate longitudinal exposure assessment, biomarkers of metal metabolism and oxidative stress, genomic susceptibility, and multiple matrices (hair, blood, urine) to better characterize the temporal and mechanistic relationships between environmental metals exposure, trace element homeostasis, and ASD. A more comprehensive understanding of these interactions may contribute to clarifying the potential role of environmental metals and trace elements homeostasis in ASD pathophysiology.

## Data Availability

No datasets were generated or analysed during the current study.
